# Supplementary material for: Single-cell analysis identifies the CNP/GC-B/cGMP axis as marker and regulator of modulated VSMCs in atherosclerosis
Source: Nat Commun. 2025 Jan 15;16:429. doi: 10.1038/s41467-024-55687-9 (PMC11735800; doi:10.1038/s41467-024-55687-9)
Supplement: Supplementary file 1 — Supplementary Information [file 41467_2024_55687_MOESM1_ESM.pdf]

Supplementary Information

Single-cell analysis identifies the CNP/GC-B/cGMP axis as marker and regulator of modulated VSMCs in atherosclerosis

Moritz Lehners[1], Hannes Schmidt[1], Maria T K Zaldivia[1], Daniel Stehle[1], Michael Krämer[1], Andreas Peter[2], Julia Adler[3], Robert Lukowski[3], Susanne Feil[1], Robert Feil[1,*]

[1] Interfakultäres Institut für Biochemie, University of Tübingen, Germany

[2] Institute for Clinical Chemistry and Pathobiochemistry, Department for Diagnostic Laboratory Medicine, University Hospital Tübingen, Germany

[3] Department of Pharmacology, Toxicology and Clinical Pharmacy, Institute of Pharmacy, University of Tübingen, Germany

[*] Corresponding author: Robert Feil, Interfakultäres Institut für Biochemie, University of Tübingen, Auf der Morgenstelle 34, 72076 Tübingen, Germany; e-mail: robert.feil@uni-tuebingen.de

Supplementary Figures

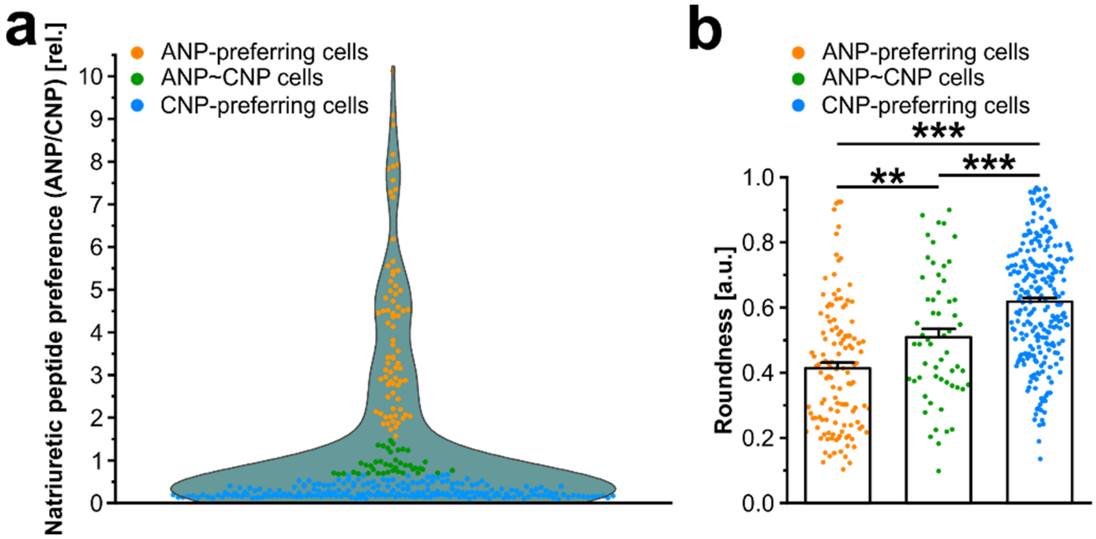

Supplementary Fig. 1 │ Detailed analysis of morphology and natriuretic peptide preference of primary VSMCs. a, Violin plot showing the distribution of natriuretic peptide preference by exact values. VSMCs responding only to ANP but not to CNP and *vice versa* were excluded ("0" and "infinity"). Classification in ANP-preferring, ANP~CNP, and CNP-preferring cells is indicated by the color code shown in panel a. For n-numbers, see Fig. 1c. **b,** Characterization of the morphology of the ANP-preferring (orange), ANP~CNP (green), and CNP-preferring (cyan) primary VSMCs. The "roundness" parameter of all ROIs used to analyze the cGMP/FRET responses of the primary VSMCs shown in Fig. 1c was assessed with Fiji. This parameter ranges from "0" for elongated cells to "1" for round cells. Each data point represents an individual ROI/cell. Data are shown as mean + SEM (for n-numbers, see Fig. 1c). Statistical significance is indicated by asterisks (** $p<0.01$; *** $p<0.001$). Source data including exact p-values and applied statistical tests are provided in the Source Data file.

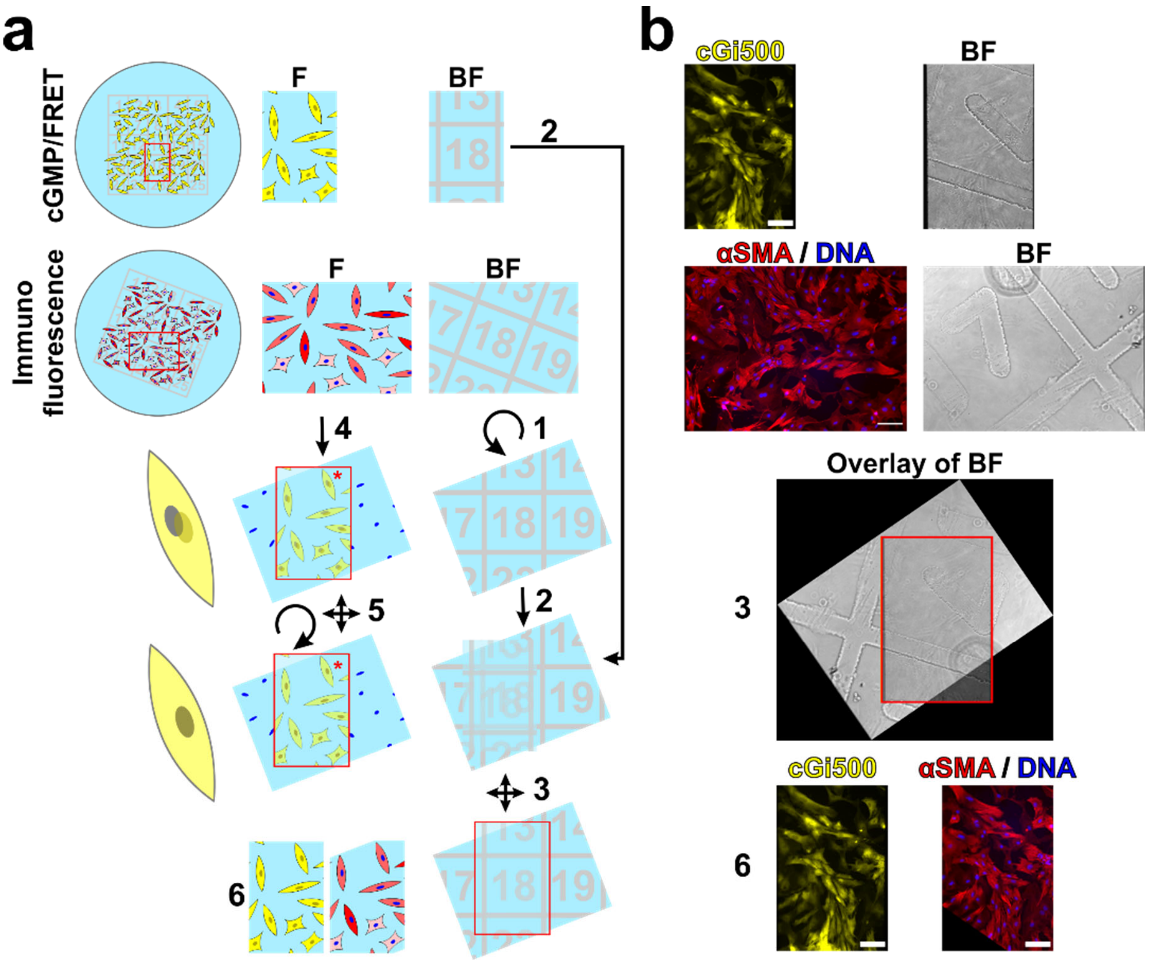

Supplementary Fig. 2 │ Mapping method to correlate cGMP/FRET measurements in individual cells with marker protein expression analyzed by immunofluorescence staining. a, Schematic step by step explanation. Cells are grown on gridded coverslips. Before starting a cGMP/FRET measurement, cells are documented by their sensor fluorescence (F, snapshot) and their position on the coverslip is assessed with a brightfield (BF) image of the grid (top). After immunofluorescence staining, the region of interest is identified via the coordinates of the grid, (1) the angle of one grid line is determined in both BF images, and the BF image of the IF staining is rotated to match both angles. (2-3) Then the grid is used to roughly align the images of the antibody staining with the images of the cGMP measurement. (4-5) Then, the YFP fluorescence image of the VSMCs obtained during the cGMP/FRET measurement is overlayed with the nuclear staining of the immunofluorescence image. The YFP fluorescence image is moved until the nuclei in both images are perfectly overlayed (a magnified image of a cell is shown left of 4 and 5). (6) Finally, all IF images are aligned with the refined settings and cropped. Now, all cells of the IF staining are at the exact same position as the cells during the cGMP/FRET measurement. **b,** Illustration of the mapping procedure shown in panel a with images from an actual cGMP/FRET measurement with subsequent immunostaining. Numbers refer to the respective steps in panel a. At the end of step 6, the positions of cells measured by cGMP/FRET imaging perfectly match the positions of the cells after immunofluorescence staining. Scale bars, 100 µm. BF, brightfield; F, fluorescence.

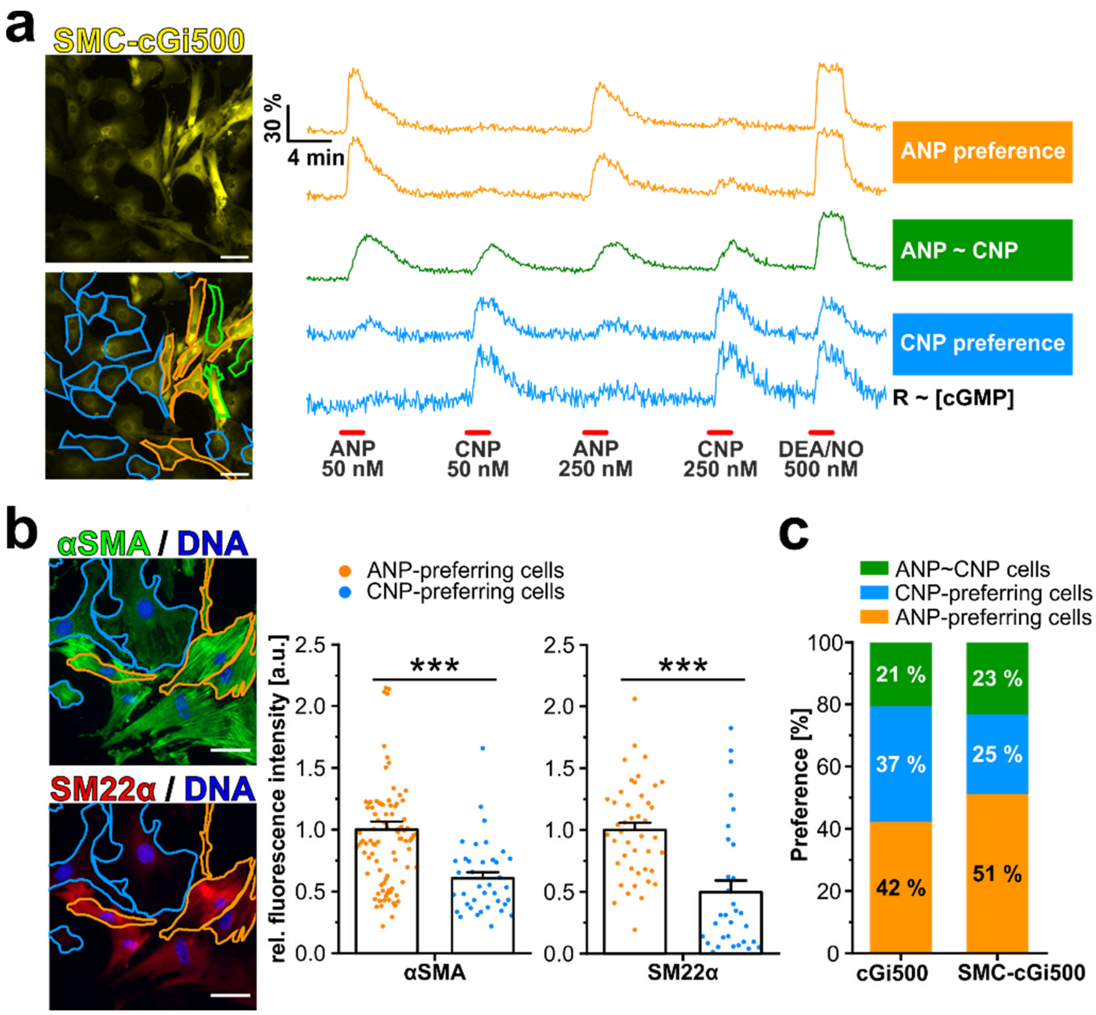

Supplementary Fig. 3 │ cGMP imaging and correlation of cell phenotype and cGMP response pattern in primary VSMCs from SMC-specific cGMP sensor mice. Cells were isolated from SMC-specific cGMP sensor mice (SMC-cGi500) and grown and imaged for cGMP as described in **Fig. 1**. **a,** Representative cGMP/FRET measurement (ratio traces R ~ [cGMP]) of individual VSMCs that were consecutively stimulated with ANP, CNP, and DEA/NO (red bars, concentrations indicated in the panel). The ratio traces represent 162 analyzed cells out of 243 recorded cells (on four coverslips from one cell isolation). Like in experiments with global sensor mice, cells were classified as "ANP-preferring" (orange, 73 cells), cells without a clear preference for ANP or CNP ("ANP~CNP", green, 42 cells), and "CNP-preferring" (cyan, 47 cells). The black scale bars indicate the time and percent change of the traces relative to baseline. The pictures on the left show cGMP sensor expressing VSMCs visualized by the YFP fluorescence of cGi500. In the lower picture, the cGMP response pattern of each cell is highlighted using the same color code as for the cGMP traces in the right panel. White scale bars, 50 µm. Results were replicated in independent experiments (see, panel c). **b,** To correlate the phenotype and cGMP response pattern of an individual cell, we have used a mapping method based on gridded coverslips. For details, see Methods section and **Supplementary Fig. 2**. Primary VSMCs from SMC-specific cGMP sensor mice were imaged for cGMP, followed by immunofluorescence staining for contractile marker proteins αSMA and SM22α. The panels show representative images and the quantitative evaluation of fluorescence intensities (normalized to the mean of ANP-preferring cells) of individual VSMCs. ANP-preferring cells are indicated by orange cell borders and data points, and CNP-preferring cells by cyan cell borders and data points. Each data point represents an individual VSMC. Data are shown as mean + SEM (αSMA: 132 analyzed cells out of 221 recorded cells on eight coverslips from two cell isolations; similar results were obtained in another independent experiment; SM22α: 75 analyzed cells out of 118 recorded cells on four

coverslips from two cell isolations). Cells classified as ANP~CNP cells are not shown and, therefore, not included in the total count of recorded cells. Statistical significance is indicated by asterisks (*** $p<0.001$). Scale bars, 50 µm. DNA was stained with Hoechst No. 33258 (blue). **c,** Comparison of ANP/CNP preference in primary VSMC cultures from global (cGi500) and SMC-specific (SMC-cGi500) cGMP sensor mice. Bars indicate the fraction of cells in each category (cGi500: 2440 analyzed cells out of 2622 recorded cells on 45 coverslips from 13 cell isolations; SMC-cGi500: 373 analyzed cells out of 627 recorded cells on 17 coverslips from 6 cell isolations). As detailed in the Methods section, cells showing a poor quality of their ratio traces or that could not be analyzed in the immunostaining due to limitations of the mapping technique were excluded from analysis. The respective numbers of analyzed cells out of total recorded cells are indicated above. Source data including exact p-values and applied statistical tests are provided in the Source Data file.

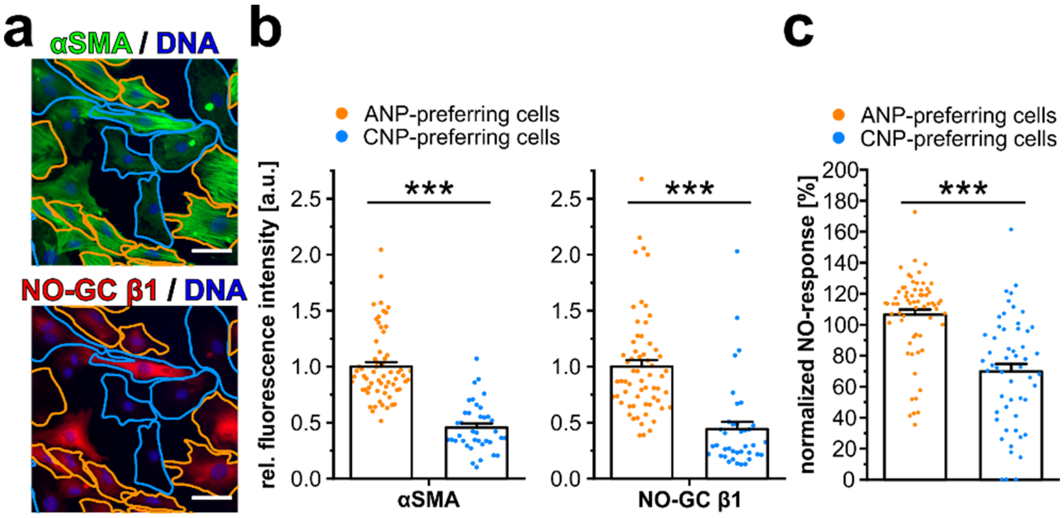

Supplementary Fig. 4 │ Analysis of NO-dependent cGMP signaling in primary VSMCs as a function of ANP/CNP preference. Cells from global cGMP sensor mice were grown and imaged for cGMP as described in **Fig. 1**. To correlate protein expression and cGMP response pattern of an individual cell, we have used a mapping method based on gridded coverslips. For details, see Methods section and **Supplementary Fig. 2**. **a, b,** Primary VSMCs were imaged for cGMP followed by immunofluorescence staining for αSMA and NO-GC β1. The panels show **(a)** representative images and **(b)** the quantitative evaluation of fluorescence intensities (normalized to the mean of ANP-preferring cells) of individual VSMCs. ANP-preferring cells are indicated by orange cell borders and data points, and CNP-preferring cells by cyan cell borders and data points. Each data point represents an individual cell. Data are shown as mean + SEM (100 cells analyzed out of 121 recorded cells on four coverslips from one cell isolation). Statistical significance is indicated by asterisks (*** $p<0.001$). Scale bars, 50 µm. DNA was stained with Hoechst No. 33258 (blue). **c,** Comparison of the NO-induced cGMP response between ANP-(orange) and CNP-preferring (cyan) cells. For each cell, the NO-induced cGMP response (peak height) was normalized to the highest cGMP response elicited by ANP or CNP, whichever was higher. Each data point represents an individual cell. Data are shown as mean + SEM (119 cells analyzed out of 121 recorded cells on four coverslips from one cell isolation). Statistical significance is indicated by asterisks (*** $p<0.001$). As detailed in the Methods section, cells showing a poor quality of their ratio traces or that could not be analyzed in the immunostaining due to limitations of the mapping technique were excluded from analysis. The respective numbers of analyzed cells out of total recorded cells are indicated above. Cells classified as ANP~CNP cells are not shown and, therefore, not included in the total count of recorded cells. Source data including exact p-values and applied statistical tests are provided in the Source Data file.

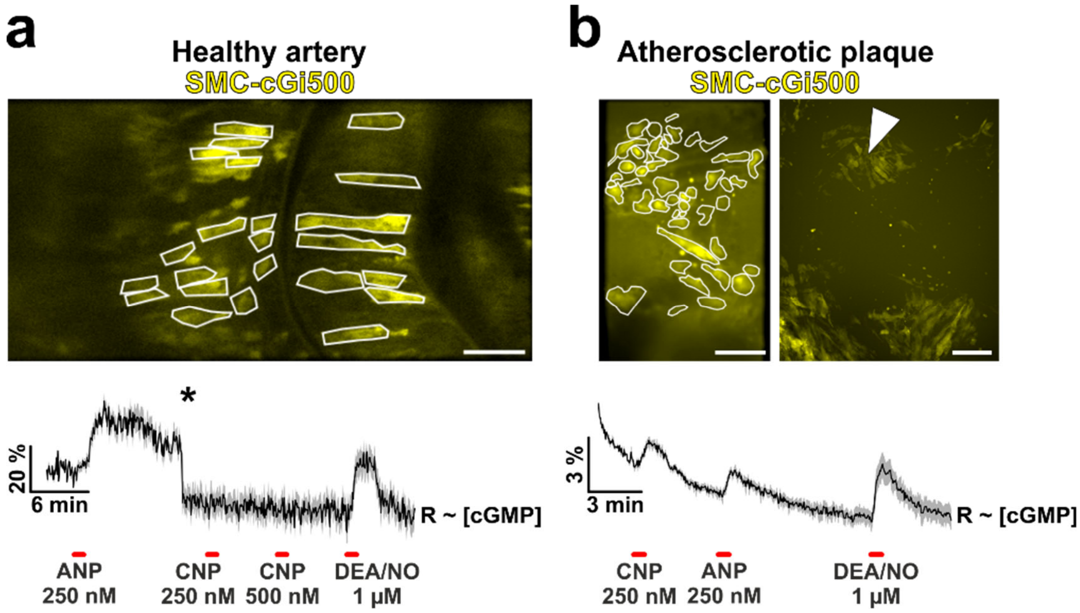

Supplementary Fig. 5 │ Analysis of cGMP responses in healthy and atherosclerotic aortae from SMC-specific cGMP sensor mice (SMC-cGi500). a, b, Representative cGMP/FRET recordings of **(a)** a healthy and **(b)** an atherosclerotic aorta that were consecutively stimulated with ANP, CNP, and DEA/NO (red bars, concentrations indicated in the panel). The black traces indicate the intracellular cGMP concentration over time (ratio trace R ~ [cGMP]). Shown are means ± SEM (grey shadow behind the trace). Healthy artery: 8 ROIs/cells with the same response pattern; atherosclerotic artery: 5 ROIs/cells with the same response pattern. The black scale bars indicate the time and percent change of the traces relative to baseline. The asterisk indicates a refocus event. The images show **(a)** a healthy aorta and (**b,** left) an atherosclerotic plaque during a cGMP/FRET measurement, and (**b,** right) a maximum intensity projection of the atherosclerotic plaque. Sensor-expressing cells are visualized by the YFP fluorescence of cGi500. ROIs/cells used for the analysis of cGMP responses are indicated by grey outlines. White scale bars in panels a and b from left to right, 20 µm, 50 µm, and 50 µm. The white arrowhead indicates the region that was measured. 31 out of 92 recorded ROIs/cells in healthy arteries (in six measurements from five aorta isolations) reacted to ANP and/or NO. 88 out of 102 recorded ROIs/cells in atherosclerotic plaques (in three measurements from one atherosclerotic aorta) could be analyzed. 20 of these ROIs/cells reacted to CNP. As detailed in the Methods section, ROIs/cells showing a poor quality of their single and/or ratio traces were excluded from analysis. The respective numbers of analyzed ROIs/cells out of total recorded ROIs/cells are indicated above. Source data are provided in the Source Data file.

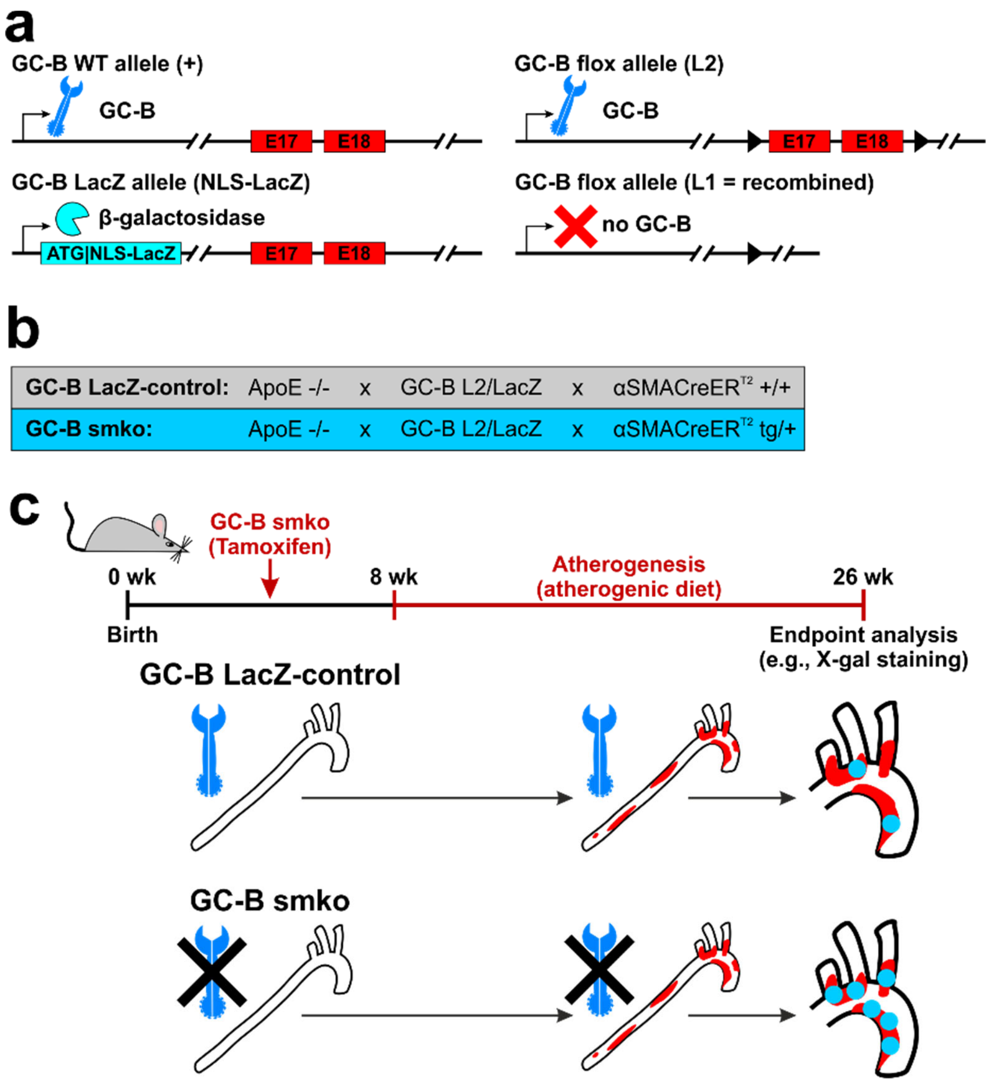

Supplementary Fig. 6 │ Generation of experimental animals to analyze the role of GC-B in VSMCs for atherosclerosis. a, Schematic representation of the different GC-B alleles of experimental mice: GC-B wildtype allele ("+", top left), GC-B LacZ allele ("NLS-LacZ", bottom left), GC-B flox allele ("L2", top right), excised GC-B flox allele after Cre-mediated recombination ("L1", bottom right). The expected gene product is indicated above each allele. The LacZ reporter gene replaces the first exon of the GC-B gene. As a nuclear localization sequence (NLS) is fused to the LacZ gene, the β-galactosidase will be located in the nucleus. Exons 17 and 18 of GC-B flox mice are flanked by loxP sites (triangle). **b,** Genotype of GC-B LacZ-control (gray) and GC-B smko (cyan) mice that were used for the analysis of atherosclerosis. **c,** Overview of the procedure to generate atherosclerotic SMC-specific GC-B knockout (GC-B smko) and control mice that express the GC-B LacZ reporter (nuclear β-galactosidase). All mice had an ApoE-deficient genetic background. The timeline of the experiment is depicted at the top. The expression of GC-B in GC-B LacZ-control and GC-B smko mice at different time points is illustrated below the timeline. Tamoxifen was injected i.p. for five consecutive days at 4 weeks of age. 8 weeks after birth, mice were fed an atherogenic diet for 18 weeks. Afterwards, mice were sacrificed, and the aorta was isolated for further analysis. The result of X-gal staining as endpoint analysis is shown at the right. Note that only the nuclei of cells are stained with X-gal by this reporter system. Wrench-like structure: GC-B, cyan dots: nuclei of X-gal+/GC-B LacZ cells, red patches: atherosclerotic plaques.

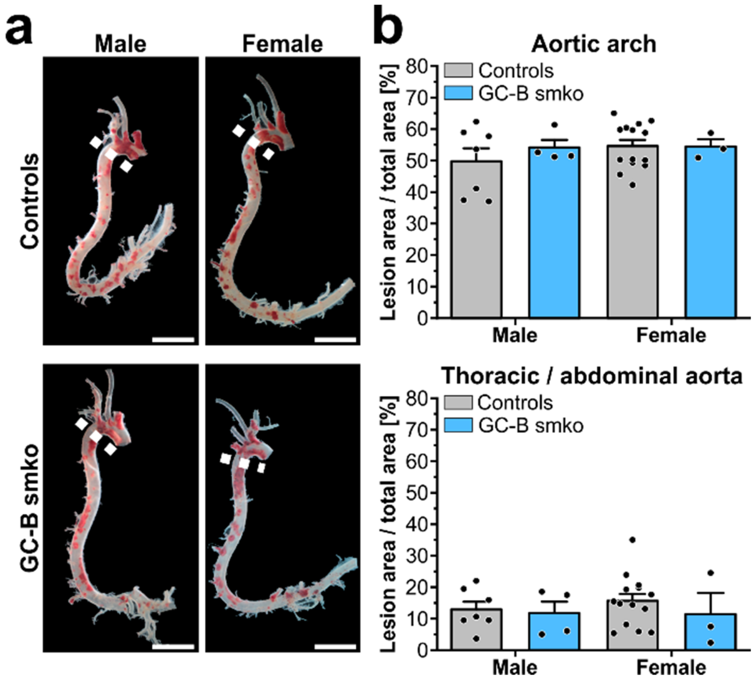

Supplementary Fig. 7 │ Detailed quantification of lesion area in atherosclerotic aortae of control and GC-B smko mice after 18 weeks of atherogenic diet. a, Representative images of atherosclerotic aortae of control and GC-B smko mice after Oil Red O staining. Atherosclerotic lesions appear red. Dashed lines indicate the transition from the aortic arch to the thoracic / abdominal aorta. Scale bars, 4 mm. **b,** Relative lesion area (percent of the respective vessel area) of different regions of the aorta from male and female control and GC-B smko mice. Each data point represents an individual aorta. Data are shown as mean + SEM (male controls: 7 aortae; male GC-B smko: 4 aortae; female controls: 14 aortae; female GC-B smko: 3 aortae). No statistically significant differences between sex or genotype were found. Source data including exact p-values and applied statistical tests are provided in the Source Data file.

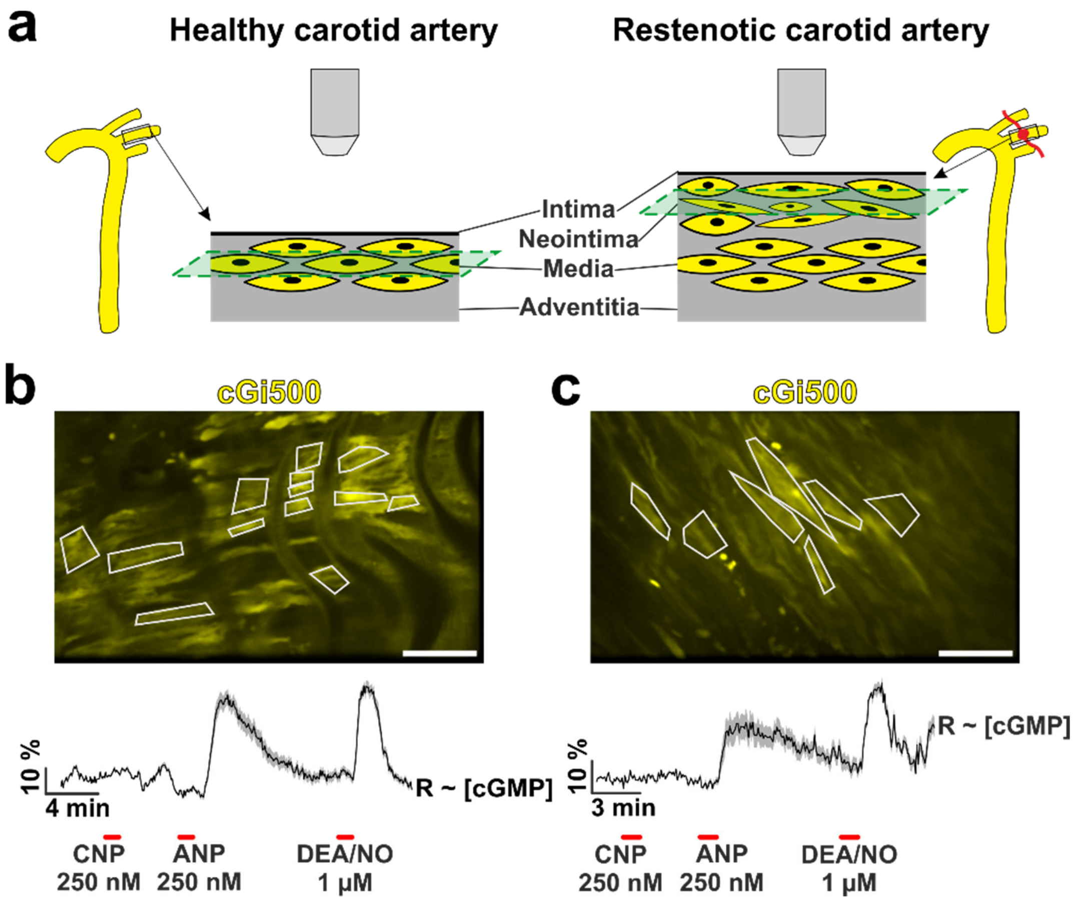

Supplementary Fig. 8 │ Analysis of cGMP responses in healthy and restenotic carotid arteries from global cGMP sensor mice. a, Schematic representation of **(left)** a healthy and **(right)** a restenotic carotid artery expressing the cGi500 sensor (yellow) as used for ex vivo cGMP/FRET measurements. The image planes are indicated in green. The ligation is indicated by a red thread. Carotid arteries were measured from the luminal side as indicated by the objective. **b, c,** Representative cGMP/FRET recordings of **(b)** a healthy and **(c)** a restenotic carotid artery that were consecutively stimulated with CNP, ANP, and DEA/NO (red bars, concentrations indicated in the panel). The black traces indicate the intracellular cGMP concentration over time (ratio trace R ~ [cGMP]). Shown are means ± SEM (grey shadow behind the trace) Healthy artery: 6 ROIs/cells with the same response pattern; restenotic artery: 4 ROIs/cells with the same response pattern. The black scale bars indicate the time and percent change of the traces relative to baseline. The images show **(b)** a healthy and **(c)** a restenotic carotid artery during a cGMP/FRET measurement. Sensor-expressing cells are visualized by the YFP fluorescence of cGi500. ROIs/cells used for the analysis of cGMP responses are indicated by grey outlines. White scale bars in panels b and c, 25 µm. 76 out of 89 recorded ROIs/cells in healthy carotid arteries (in six measurements from five aorta isolations) reacted to ANP and/or NO. All 12 recorded ROIs/cells in restenotic carotid arteries (in two measurements from two aorta isolations) reacted to ANP and/or NO. None of the analyzed ROIs/cells responded to CNP. As detailed in the Methods section, cells showing a poor quality of their single and/or ratio traces were excluded from analysis. The respective numbers of analyzed ROIs/cells out of total recorded ROIs/cells are indicated above. Source data are provided in the Source Data file.

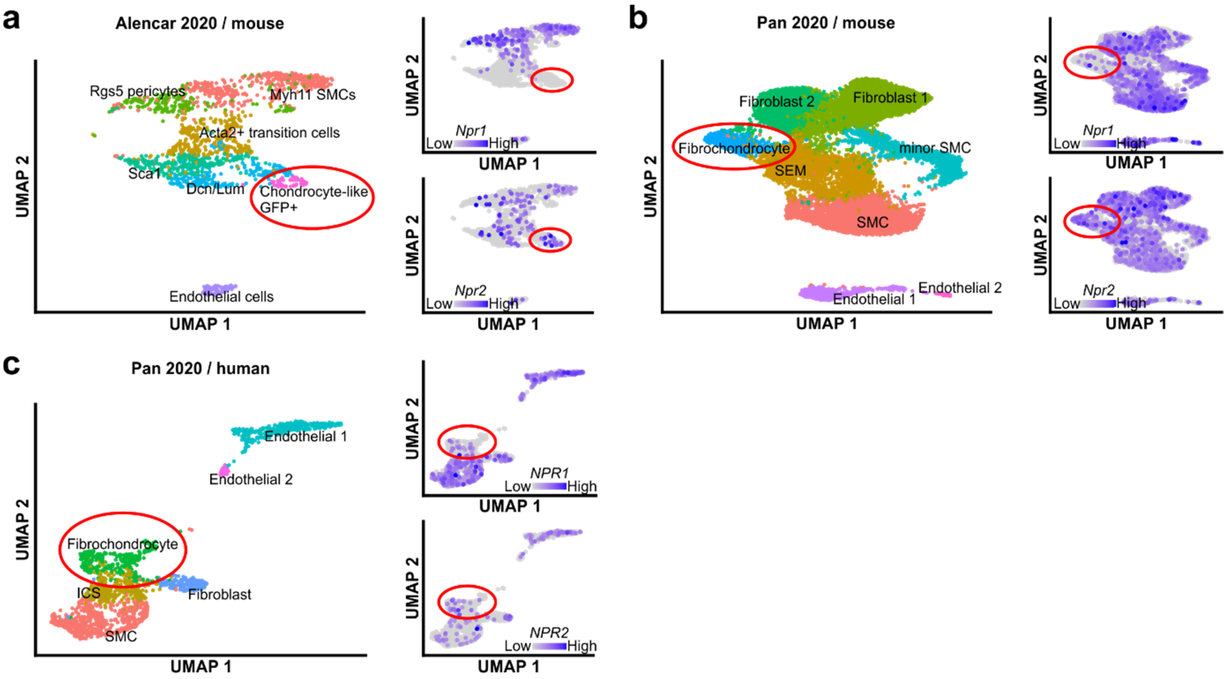

d

Original publication	Source	Cluster name	Npr1 expressing cells (% of cells in cluster)	Npr2 expressing cells (% of cells in cluster
Alencar 2020	Mouse	Myh11 SMCs	40.7%	10.5%
		Chondrocyte-like GFP+	0.0%	19.6%
Pan 2020	Mouse	SMC	48.1%	12.7%
		Fibrochondrocyte	1.9%	21.8%
Pan 2020	Human	SMC	37.7%	4.9%
		Fibrochondrocyte	3.2%	6.4%

Supplementary Fig. 9 | Characterization of Npr1 and Npr2 expression in published murine and human scRNA-seq datasets acquired from PlaqView. UMAP visualization of various cell clusters from atherosclerotic plaques (left panels) and their Npr1 (GC-A) and Npr2 (GC-B) mRNA expression (right panels). Two murine datasets[78,79] **(a, b)** and one human dataset[78] **(c)** are shown. Processed datasets were downloaded from the PlaqView portal and authors' annotations of the cell clusters were used. Cell clusters that are not relevant to our study (e.g., immune cells) are not shown. Please consult the original publications for a detailed description of the annotated cell clusters. The expression level is illustrated by a color scale. Each dot represents a single cell. Red circles highlight modulated SMC clusters with high Npr2 and low Npr1 expression. SMC, smooth muscle cell; SEM, stem cell/endothelial cell/monocyte; ICS, intermediate cell state. **d,** Expression of Npr1 (GC-A) and Npr2 (GC-B) mRNA in contractile SMCs and modulated SMC clusters contained in the published datasets shown in panels a-c. Datasets were inspected for the percentage of cells that expressed Npr1 or Npr2 mRNA in each cluster. Accession codes to the underlying data from the PlaqView portal are provided in the Source Data file.

Supplementary Table

Supplementary Table 1: Serum lipid profiles and physiological parameters of 26-week-old atherosclerotic control and GC-B smko mice after 18 weeks atherogenic diet. Data are shown as mean ± SEM. Statistical significance vs. male mice of the same genotype is indicated by asterisks (* p<0.05; ** p<0.01; *** p<0.001). There were no statistically significant differences between genotypes of the same sex. HDL, high-density lipoprotein; LDL, low-density lipoprotein. Source data including exact p-values and applied statistical tests are provided in the Source Data file.

	Males		Females	
**Parameter**	**Control**	**GC-B smko**	**Control**	**GC-B smko**
	(n = 9)	(n = 4)	(n = 16)	(n = 5)
Total cholesterol [mg/dL]	1263 ± 91	1269 ± 38	778 ± 64***	842 ± 75**
Triglyceride [mg/dL]	79 ± 10	95 ± 25	84 ± 9	85 ± 24
HDL [mg/dL]	10 ± 1	13 ± 1	9 ± 1	9 ± 2
LDL [mg/dL]	291 ± 40	289 ± 16	155 ± 16**	143 ± 23**
	(n = 9)	(n = 4)	(n = 16)	(n = 6)
Body weight [g]	35.5 ± 1.5	34.5 ± 0.9	26.3 ± 0.7***	23.4 ± 0.8***
Heart-to-body weight [mg/g]	7.9 ± 0.2	6.9 ± 0.6	6.8 ± 0.2*	7.0 ± 0.3
Kidney-to-body weight [mg/g]	16.2 ± 0.8	17.4 ± 1.1	15.1 ± 0.5	14.3 ± 1.3
