# Supplementary material for: Single-cell analysis identifies the CNP/GC-B/cGMP axis as marker and regulator of modulated VSMCs in atherosclerosis
Source: Nat Commun. 2025 Jan 15;16:429. doi: 10.1038/s41467-024-55687-9 (PMC11735800; doi:10.1038/s41467-024-55687-9)
Supplement: Supplementary file 3 — Description of Additional Supplementary Files [file 41467_2024_55687_MOESM3_ESM.pdf]

<u>Description of Additional Supplementary Files</u>

Supplementary Movie 1 | Visualization of cGMP signaling diversity in primary VSMCs in real time at the single-cell level. cGMP/FRET imaging of primary VSMCs from global cGMP sensor mice recorded at 0.2 Hz. VSMCs were stimulated with ANP, CNP, and DEA/NO (red bars, concentrations indicated in the right panel). In the movie (left), a CNP-preferring cell (cyan) and an adjacent ANP-preferring cell (orange) are highlighted. Their ratio traces reflecting the intracellular cGMP concentration (R ~ [cGMP]) are depicted at the right. Drug applications are indicated by red bars (right) and as labels during the movie (top left corner). cGMP signals (R ~ [cGMP]) of the cells are shown with a "fire" look up table (low cGMP: purple/blue, high cGMP: yellow/white) and superimposed with an image of the cells (sensor fluorescence, grey) that was acquired before the measurement. The video runs at 20 fps (100-times faster).

Supplementary Movie 2 | Visualization of cGMP signals in atherosclerotic plaques in real time at the single-cell level. cGMP/FRET imaging of the atherosclerotic plaque shown in Fig. 5, recorded at 0.2 Hz. In the right panel, the cGMP signals measured in an exemplary cell are depicted. The plaque was successively stimulated with CNP, ANP, and DEA/NO. Drug applications are indicated by red bars (right) and as labels during the movie (top left corner). Cyan and yellow traces show CFP and YFP fluorescence of the sensor, respectively. The black trace indicates the intracellular cGMP concentration over time (ratio trace of CFP/YFP or R ~ [cGMP]). At the beginning of the movie, a 3D reconstruction of the plaque (sensor fluorescence, yellow) is shown. In the movie, cGMP signals (R ~ [cGMP]) are shown with a "fire" look up table (low cGMP: purple/blue, high cGMP: yellow/white). The video runs at 20 fps (100-times faster).
