# Supplementary material for: Single-cell analysis identifies the CNP/GC-B/cGMP axis as marker and regulator of modulated VSMCs in atherosclerosis
Source: Nat Commun. 2025 Jan 15;16:429. doi: 10.1038/s41467-024-55687-9 (PMC11735800; doi:10.1038/s41467-024-55687-9)
Supplement: Supplementary file 6 — Reporting Summary [file 41467_2024_55687_MOESM6_ESM.pdf]

nature portfolio

Corresponding author(s): Robert Feil

Last updated by author(s): Nov 27, 2024

Reporting Summary

Nature Portfolio wishes to improve the reproducibility of the work that we publish. This form provides structure for consistency and transparency in reporting. For further information on Nature Portfolio policies, see our Editorial Policies and the Editorial Policy Checklist.

Statistics

For all statistical analyses, confirm that the following items are present in the figure legend, table legend, main text, or Methods section.

n/a	Confirmed	
☐	☒	The exact sample size (n) for each experimental group/condition, given as a discrete number and unit of measurement
☐	☒	A statement on whether measurements were taken from distinct samples or whether the same sample was measured repeatedly
☐	☒	The statistical test(s) used AND whether they are one- or two-sided<br>*Only common tests should be described solely by name; describe more complex techniques in the Methods section.*
☒	☐	A description of all covariates tested
☐	☒	A description of any assumptions or corrections, such as tests of normality and adjustment for multiple comparisons
☐	☒	A full description of the statistical parameters including central tendency (e.g. means) or other basic estimates (e.g. regression coefficient) AND variation (e.g. standard deviation) or associated estimates of uncertainty (e.g. confidence intervals)
☐	☒	For null hypothesis testing, the test statistic (e.g. F, t, r) with confidence intervals, effect sizes, degrees of freedom and P value noted<br>*Give P values as exact values whenever suitable.*
☒	☐	For Bayesian analysis, information on the choice of priors and Markov chain Monte Carlo settings
☒	☐	For hierarchical and complex designs, identification of the appropriate level for tests and full reporting of outcomes
☒	☐	Estimates of effect sizes (e.g. Cohen's d, Pearson's r), indicating how they were calculated

Our web collection on statistics for biologists contains articles on many of the points above.

Software and code

Policy information about availability of computer code

Data collection	The FRET imaging data were acquired by VisiView software (Visitron Systems, latest version 6.0.0.13) or arivis Browser 2D software (arivis GmbH).
Data analysis	Offline image analysis was done using Fiji (latest version 1.54k) with the following plugins: MultiStackReg (latest version 1.46.5), BIOP SimpleColorBalance (latest release 11 Oct 2023). For further data evaluation, Microsoft Excel 365 (Microsoft) and Origin Pro 2019 (OriginLab) were used. For processing and analysis of scRNAseq data, the Cell Ranger software (10x Genomics, version 3.0.2) and the R package Seurat (latest version 5.1.0) together with RStudio (Posit Software, latest version 2024.09.1) and R (latest version 4.4.2) were used.

For manuscripts utilizing custom algorithms or software that are central to the research but not yet described in published literature, software must be made available to editors and reviewers. We strongly encourage code deposition in a community repository (e.g. GitHub). See the Nature Portfolio guidelines for submitting code & software for further information.

Data

Policy information about availability of data

All manuscripts must include a data availability statement. This statement should provide the following information, where applicable:

- Accession codes, unique identifiers, or web links for publicly available datasets
- A description of any restrictions on data availability
- For clinical datasets or third party data, please ensure that the statement adheres to our policy

Source data are provided with this paper. The raw scRNA-seq data of cultured primary aortic VSMCs, generated in this study, has been deposited in the Gene Expression Omnibus (GEO) database under accession code GSE282944. The processed version of this data is available in the PlaqView portal (https://www.plaqview.com/) under the accession code Lehners_2024. The scRNA-seq data shown in Supplementary Fig. 9 is available in the PlaqView portal (https://www.plaqview.com/) under accession codes Alencar_2020_dual, Pan_2020_mouse, and Pan_2020.

Research involving human participants, their data, or biological material

Policy information about studies with human participants or human data. See also policy information about sex, gender (identity/presentation), and sexual orientation and race, ethnicity and racism.

Reporting on sex and gender	N/A
Reporting on race, ethnicity, or other socially relevant groupings	N/A
Population characteristics	N/A
Recruitment	N/A
Ethics oversight	N/A

Note that full information on the approval of the study protocol must also be provided in the manuscript.

Field-specific reporting

Please select the one below that is the best fit for your research. If you are not sure, read the appropriate sections before making your selection.

☒ Life sciences ☐ Behavioural & social sciences ☐ Ecological, evolutionary & environmental sciences

For a reference copy of the document with all sections, see nature.com/documents/nr-reporting-summary-flat.pdf

Life sciences study design

All studies must disclose on these points even when the disclosure is negative.

Sample size	Sample sizes were chosen based on data from previous publications, which were sufficient for statistical analysis.
Data exclusions	Cells that showed a poor quality of their ratio traces or that could not be analyzed in the immunostaining due to limitations of the mapping technique were excluded from analysis, as described in Methods. Exclusion criteria have been defined prior to experiments.
Replication	Experiments were successfully replicated and numbers of replication were stated in all relevant figure legends.
Randomization	No randomization of mice was applied because all mice received the same treatment. Mice analyzed were litter mates of both sexes. Allocation of mice to experimental groups was solely based on their genotype.
Blinding	Investigators were blinded to mouse genotypes and sex during experiments. Data reported for mouse experiments are not subjective but rather based on quantitative FRET imaging and analysis of atherosclerotic lesions.

Reporting for specific materials, systems and methods

We require information from authors about some types of materials, experimental systems and methods used in many studies. Here, indicate whether each material, system or method listed is relevant to your study. If you are not sure if a list item applies to your research, read the appropriate section before selecting a response.

Materials & experimental systems

n/a	Involved in the study
☐	☒ Antibodies
☒	☐ Eukaryotic cell lines
☒	☐ Palaeontology and archaeology
☐	☒ Animals and other organisms
☒	☐ Clinical data
☒	☐ Dual use research of concern
☒	☐ Plants

Methods

n/a	Involved in the study
☒	☐ ChIP-seq
☒	☐ Flow cytometry
☒	☐ MRI-based neuroimaging

Antibodies

Antibodies used

Antibodies directed against the following proteins were used for Western blotting (WB), immunofluorescence (IF), and immunohistochemistry (IHC):
SM22α (1:1000 WB, 1:500 IF, rabbit, #ab14106, Abcam)
αSMA (1:2000 IHC, rabbit, #ab124964, Abcam)
MAC-2 (1:200 IHC, rat, #Cl8942, Cedarlane)
PDGFRα (1:500 IF, rabbit, #3174, Cell Signaling)
GAPDH (1:1000 WB, rabbit, #2118, Cell Signaling)
horse radish peroxidase (HRP)-conjugated anti-rabbit antibody (1:5000 WB, goat, #70749, Cell Signaling)
HRP-conjugated anti-guinea pig antibody (1:10,000 WB, donkey, #706-035-148, Dianova)
Alexa Fluor 488-conjugated anti-mouse antibody (1:500 IF, goat, #A11029, Life Technologies)
Alexa Fluor 555-conjugated anti-mouse antibody (1:500 IF, goat, #A21424, Life Technologies)
Alexa Fluor 488-conjugated anti-rabbit antibody (1:500 IF, goat, #A11008, Life Technologies)
Alexa Fluor 555-conjugated anti-rabbit antibody (1:500 IF, goat, #A21428, Life Technologies)
S100A4 (1:300 IF, rabbit, #07-2274, Millipore)
osteopontin (1:100 IHC, goat, #AF808, R&D Systems)
αSMA (1:500 IF, mouse, #A2547, Sigma-Aldrich)
biotinylated anti-goat antibody (1:250, horse, #BA-9500, Vector Laboratories)
biotinylated anti-rabbit antibody (1:250 IHC, goat, #BA-1000, Vector Laboratories)
biotinylated anti-rat antibody (1:250 IHC, rabbit, #BA-4001, Vector Laboratories)
GC-B antibody (1:5000 WB, guinea pig) was from Hannes Schmidt (Ref 66)
NO-GCβ1 1A antibody (1:800 IF, rabbit) and NO-GCβ1 2A antibody (1:10,000 WB, rabbit) were a gift from Andreas Friebe (Ref 96)
cGKI antibody (1:5000 WB, rabbit) was generated in the Feil laboratory (Ref 97)

Validation

Commercial antibodies used in the study were validated by the manufacturers.. All customized antibodies had been validated in experiments using knockout mice.

Animals and other research organisms

Policy information about studies involving animals; ARRIVE guidelines recommended for reporting animal research, and Sex and Gender in Research

Laboratory animals

Description of experimental mice can be found in the relevant figure legends and the Methods section.
Mice were housed with ad libitum access to food and water with a 12 h light/dark cycle. Room temperature was set to 22 °C and humidity kept at 50-60 %.

Wild animals

The study did not involve wild animals.

Reporting on sex

Sex is reported in the Methods section and in the relevant figure legends. Female and male mice were used in all experiments.

Field-collected samples

The study did not involve any samples collected from field.

Ethics oversight

Regierungspräsidium Tübingen, Germany

Note that full information on the approval of the study protocol must also be provided in the manuscript.

Plants

Seed stocks	N/A
Novel plant genotypes	N/A
Authentication	N/A
